# Piloting a Health System Performance Assessment for Germany - insights from trend and equity analyses

**DOI:** 10.1093/eurpub/ckac131.305

**Published:** 2022-10-25

**Authors:** K Achstetter, M Blümel, P Hengel, V Schwarzbach, R Busse

**Affiliations:** Department of Health Care Management, Technische Universität Berlin, Berlin, Germany

## Abstract

**Background:**

Health System Performance Assessment (HSPA) is a tool for the evaluation of the performance and efficiency of a health system and can be used for evidence-based policymaking. For the first time, a country specific HSPA for the German health system is piloted with a focus on trend and equity analyses.

**Methods:**

Based on the conceptual framework developed in a feasibility study, the pilot study in Germany has being conducted between 2020 and 2023. The framework includes 90 internationally compatible indicators, each of which can be assigned to one of nine dimensions (e.g., access, quality, population health, responsiveness, efficiency). The aim of this pilot study is to conduct trend analyses over the period 2000-2020 and equity analyses (e.g., age, gender, region, education). Data from 56 different national and international secondary data sources (e.g., epidemiologic registry data, claims data, and survey data) were collected, analyzed, and compiled in a report.

**Results:**

In total, 84 of the 90 indicators could be analyzed with data for Germany. The indicators (e.g., access to acute care, 30-day mortality, amenable mortality rate) are prepared as a trend analysis for Germany for up to 20 years and with regard to various equity aspects. Most indicators can be presented in international comparison with eight selected European countries (e.g., Denmark, France). Furthermore, recommendations were derived to improve the availability and/or quality of data.

**Conclusions:**

The first German HSPA pilot study provides valuable insights into the performance of the health system. The results based on the analysis of the 90 indicators are an important basis for identifying inequities and needs for improvement. In the future, the lessons learned from the pilot study can be helpful for a permanent implementation of a German HSPA. However, the fragmented data structure in Germany will be a future challenge.

**Key messages:**

• The first country specific health system performance assessment for the German health system was conducted as a pilot study.

• Trends over time, inequities, and needs for improvement can be derived from the analysis of 90 indicators.

